# Effects of the dietary zinc source and vitamin E level on live weight and carcass yield and meat quality in male broilers reared under chronic cyclic heat stress conditions in the finisher phase

**DOI:** 10.3389/fphys.2022.992689

**Published:** 2022-10-06

**Authors:** Annatachja De Grande, Richard Ducatelle, Saskia Leleu, Christof Rapp, Cibele Torres, Massimiliano Petracci, Stefaan De Smet, Joris Michiels, Freddy Haesebrouck, Filip Van Immerseel, Evelyne Delezie

**Affiliations:** ^1^ Research Institute for Agriculture, Fisheries and Food (ILVO), Merelbeke, Belgium; ^2^ Department of Pathobiology, Pharmacology and Zoological Medicine, Ghent University, Merelbeke, Belgium; ^3^ Zinpro Corporation, Boxmeer, Netherlands; ^4^ Department of Agricultural and Food Sciences, Alma Mater Studiorum, University of Bologna, Cesena, Italy; ^5^ Department of Animal Sciences and Aquatic Ecology, Ghent University, Ghent, Belgium

**Keywords:** zinc, vitamin E, broiler, carcass yield, meat quality, heat stress

## Abstract

The objective of this study was to evaluate the effect of the interaction of the zinc source (ZnSO_4_ vs. zinc amino acid complex) and vitamin E level (50 IU/kg vs. 100 IU/kg) on meat yield and quality in broilers subjected to chronic cyclic heat stress in the finisher phase. A total of 1224 one-day-old male Ross 308 broilers were randomly distributed among four dietary treatments. Each treatment contained nine replicates of 34 birds, housed in floor pens in a temperature- and lighting-controlled room. Treatments were organized in a 2 × 2 factorial arrangement: two sources of zinc, 60 mg/kg of Zn as ZnSO_4_ or 60 mg/kg of Zn as zinc amino acid complexes (ZnAA), combined with two levels of vitamin E (50 or 100 IU/kg). From day 28 until day 37 (finisher phase), all birds were subjected to chronic cyclic heat stress (32 ± 2°C for 6 h daily). In the present study, it was observed that replacing ZnSO_4_ with ZnAA increased breast meat weight and yield of broilers reared under chronic cyclic heat stress conditions, whereas total slaughter yield was not affected. Moreover, it was observed that replacing ZnSO_4_ with ZnAA resulted in breast meat with a lower drip and thawing loss and a higher marinade uptake. In conclusion, replacing ZnSO_4_ with more readily available ZnAA can improve breast meat yield and increase the water-holding capacity of breast meat of broilers exposed to chronic cyclic heat stress at the end of the production cycle. However, as no thermoneutral group was included in the present study, the observed effects of the zinc source cannot be generalized as a solution for heat stress. Moreover, the beneficial effects of ZnAA on breast meat yield and quality seem to be independent of the vitamin E level, and increasing vitamin E level has no additional beneficial effects.

## 1 Introduction

Heat stress is a major concern in poultry production because it has a profound effect on animal health and performance. Modern broiler breeds display reduced heat tolerance because of a lack of sweat glands and the high metabolism associated with low feed conversion and rapid growth (Lara and Rostagno, 2013; He et al., 2018). Moreover, chronic heat stress leads to the deterioration of meat quality by changing the aerobic metabolism and by increasing glycolysis and fat deposition (Petracci et al., 2015; Lu et al., 2017; Wang et al., 2017). Consequently, the meat from broilers reared under high environmental temperatures is characterized by a pale color, low water-holding capacity (WHC), and therefore also increased cook and drip losses (Wang et al., 2017). The impaired WHC is detrimental to the valorization of broiler meat which is further processed by marination, tumbling, and cooking (Zaboli et al., 2019). Supplementation of vitamin A, C, and E can improve heat tolerance ability and animal performance during heat stress (Khan et al., 2011; Khan et al., 2012; Rehman et al., 2017). Some antioxidant minerals, including chromium (Akbari and Torki, 2014; Attia et al., 2017), selenium (Habibian et al., 2015), and zinc, (Sahin et al., 2009; Chand et al., 2014) are also used to prevent negative effects of heat stress. Zinc is an essential component of many enzymes, and it has both structural and catalytic functions in metalloenzymes. Furthermore, zinc is required for normal immune function as well as proper skeletal development and maintenance (Sahin et al., 2009). One of the most important functions of zinc is its antioxidant role and its participation in the antioxidant defense system. An increased level of reactive oxygen species is one of the main causes of decreased meat quality due to heat stress (Zaboli et al., 2019). In broiler diets, ZnSO_4_ and ZnO are two of the main inorganic zinc sources. There are also organic zinc sources available that are characterized by improved bioavailability (Star et al., 2012; Swiatkiewicz et al., 2014). A more readily available zinc source might be more efficient in reducing the adverse effects of stressors in broiler production, such as heat stress. Both zinc and vitamin E are frequently used antioxidants to alleviate the negative impact of heat stress; however, vitamin E is expensive, and lower levels may be sufficient in combination with a more readily available zinc source. To the best of our knowledge, there is no information available concerning the interaction of zinc sources, as opposed to different zinc levels, on meat quality of broilers subjected to a temperature challenge, and on the interaction with the vitamin E level. Therefore, the objective of this study was to evaluate the effect of the interaction of the zinc source (ZnSO_4_ vs. zinc amino acid complex) and vitamin E level (50 vs. 100 IU) on meat quality and yield of broilers exposed to chronic cyclic heat stress in the finisher phase.

## 2 Materials and methods

All experimental procedures in this study were in compliance with the European guidelines for the care and use of animals in research (Directive 2010: 63: EU) and were approved by the Ethical Committee of the Research Institute for Agriculture, Fisheries and Food (ILVO), Merelbeke, Belgium, under authorization number 2017: 308.

### 2.1 Experimental design, animals, and diets

A total of 1224 one-day-old male Ross 308 broilers (Belgabroed, Merksplas, Belgium) were randomly allocated to 36 floor pens (9 pens per treatment and 34 broilers per pen) in an alternating block design, with one replicate per treatment in each block. Up to day 7, the broilers were subjected to a light schedule of 23 hours of light and 1 hour of darkness. From day 7 onwards, the animals were subjected to a light schedule of 18 hours of light and 6 hours of darkness. The temperature was kept at 29°C during the first week of the experiment and reduced thereafter until a final temperature of 22°C was reached at day 28. From day 28 until day 37 (slaughter age), the temperature and relative humidity (RH) in the stable were raised up to 32 ± 2°C and 55–65%, respectively, for 3 h and subsequently maintained for 6 h before cooling down again to the initial temperature of 22°C. Heat stress was confirmed empirically on randomly selected birds from different pens equally divided over treatments by measuring the rectal temperature, in order to confirm that the temperature was raised above the physiological range of 41.2–42.2°C. The house was equipped with a dynamic ventilation system with an air entrance on one side and air extraction on the other side. The temperature and RH were constantly monitored and steered by the adjustment of the heating system and ventilation rate. Humidity was increased by nebulizing water via water nozzles and decreased by increasing the ventilation rate.

Dietary treatments were organized in a 2 × 2 factorial design with two sources of zinc (Zn). Treatments contained equal amounts of elemental zinc (60 mg/kg), either originating from ZnSO_4_ (ZnSO_4_.7H2O; containing 22% of elemental zinc, Sigma-Aldrich, St. Louis, United States) or originating as zinc amino acid complexes (ZnAA; containing 10% of elemental zinc, Availa®Zn, Zinpro Corporation, Eden Prairie, United States), and two levels of vitamin E (50 or 100 IU/kg; dl-α-tocopheryl acetate). The dietary treatments were provided in a wheat–rye-based diet (Table 1). Availa®Zn is a zinc chelate based on single amino acids from hydrolyzed soy protein and zinc bound in a one-to-one molar ratio. All dietary treatments contained equal zinc levels that comply with the dietary needs as described by NRC (1994). A vitamin E level of 50 IU/kg is recommended in the Ross 308 manual (Aviagen, 2018) and was set at the standard dose, and 100 IU/kg was selected as the elevated level of vitamin E. The total levels of zinc included in the experimental diets were determined using inductively coupled plasma atomic emission spectroscopy with a method derived from NEN 15763, ISO 21033, and ISO 27085 at the ECCA laboratory (Merelbeke, Belgium) (Table 2). The total levels of vitamin E included in the experimental diets were determined according to Claeys et al. (2016) (Table 2). A three-phase feeding scheme was applied, and dietary treatments were applied in all phases. The starter diet was fed from day 0 up to day 10 and was provided as a crumble. The grower and finisher diets were fed as pellets from day 10 up to day 28 and from day 28 up to day 37, respectively. Feed and drinking water were provided *ad libitum*.

**TABLE 1:** Dietary composition of the diets.

	Starter diet	Grower diet	Finisher diet
Ingredient (%)			
Wheat	48.54	54.45	57.65
Rye	5.00	5.00	5.00
Soybean meal (48%)	30.18	24.39	21.61
Soybeans^*^	7.50	7.50	7.42
Rapeseed meal	2.00	2.00	2.00
Animal fat	2.50	2.60	2.70
Soy oil	1.00	1.00	1.00
Vitamin + trace mineral mix^§^	1.000	1.000	1.000
CaCO_3_	0.707	0.788	0.702
Di-Ca-phosphate	0.737	0.456	0.206
NaCl	0.272	0.235	0.278
Na-bicarbonate	0.104	0.145	0.087
L-Lys-HCl	0.134	0.144	0.121
DL-Methonine	0.260	0.213	0.172
L-Threonine	0.064	0.056	0.040
Phytase^‡^	0.020	0.020	0.020
Calculated nutrient composition			
Crude protein (%)^†^	23.00	21.00	20.00
Crude fat (%)^†^	6.46	6.41	6.50
Non-soluble polysaccharides (%)	14.66	14.21	13.98
Metabolizable energy (MJ/kg)	11.00	11.25	11.46
Dig. lysine (%)	1.15	1.03	0.95
Dig. methionine + cysteine (%)	0.86	0.77	0.71
Dig. threonine (%)	0.75	0.67	0.62
Dig. valine (%)	0.89	0.81	0.76
Ca (%)	0.85	0.80	0.70
Available P (%)	0.40	0.35	0.31
NaCl + KCl (mEq/kg)	267	247	213
Linoleic acid (18:2) (%)	2.10	2.08	2.06
Analyzed nutrient composition			
Crude protein	23.67	22.09	20.90
Crude fat	6.33	6.56	6.49
Crude ash	5.21	4.75	4.46

^§^Provided per kg of diet: vitamin A (retinylacetate 3a672a), 10,000 IU; vitamin D3 (E671), 3000 IU; vitamin E (dl-α-tocopherol acetate), 50 IU (T1+T2) or 100 IU (T3+T4); vitamin K, 2.5 mg; vitamin B1 (thiamine mononitrate), 2 mg; riboflavin, 5 mg; calcium D-pantothenate, 15 mg; vitamin B6, 4 mg; vitamin B12, 0.025 mg; niacinamide, 30 mg; folic acid, 1 mg; biotin, 0.2 mg; choline (choline chloride), 689.7 mg; Cu (CuSO_4_._5_H_2_O), 20 mg; Mn (MnSO_4_.H_2_O), 95.9 mg; Fe (FeSO_4_.H_2_O), 49.2 mg; I (KI), 1.2 mg; Se (Na_2_SeO_3_), 0.4 mg; HSepioliet, 7.0 mg; propylgallate, 2.0 mg; BHT, 3.0 mg; Zn (T1+T3, ZnSO_4_._7_H_2_O, and T2+T4, Availa®Zn), 60 mg.

^‡^Ronozyme^®^ NP. 10 000 FYT/g.

**TABLE 2:** Analyzed zinc and vitamin E concentrations in the different dietary treatments for broilers.

Treatment	Added zinc or vitamin E level		Analyzed zinc level			Analyzed vitamin E level		
	Added zinc source and concentration (mg/kg)	Added VE level (IU/kg)	Starter	Grower	Finisher	Starter	Grower	finisher
1	60 mg/kg ZnSO_4_	50	94	88	90	73	82	67
2	60 mg/kg ZnAA	50	91	90	91	67	86	69
3	60 mg/kg ZnSO_4_	100	89	91	89	121	145	105
4	60 mg/kg ZnAA	100	90	98	90	113	119	104

ZnAA, zinc amino acid complex; Vit E, vitamin E, dl-α-tocopheryl acetate.

### 2.2 Slaughter yield and meat quality analysis

**FIGURE 1:**
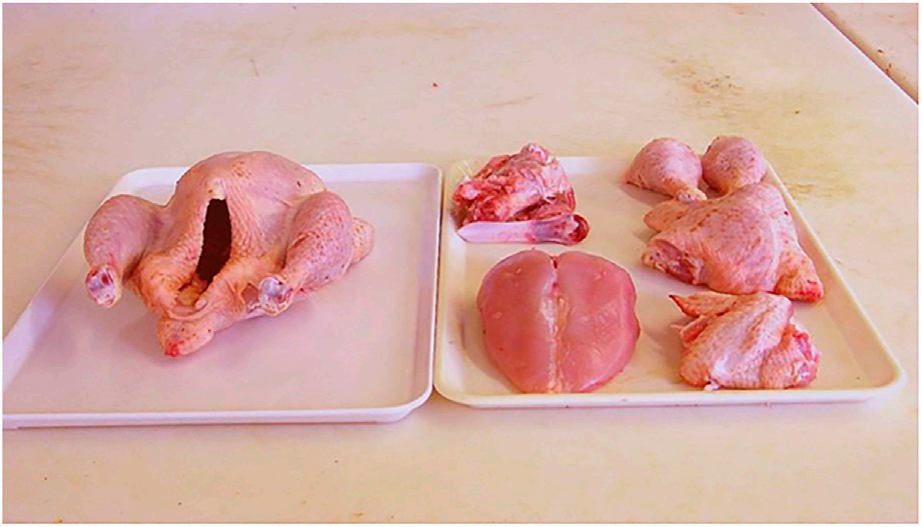
Illustration of dissected carcass to determine slaughter yield.

On day 37, three broilers per pen were selected, and live weight was determined by weighing the animals on a scale suited for animal use before transport to the slaughterhouse, where they were commercially slaughtered. Carcasses were immediately chilled after processing. Slaughter yield was determined approximately 24 h after slaughter (108 birds in total, 27 from each treatment group). The broilers were manually dissected by trained personnel to determine carcass, wing, leg (thigh and drumstick) and breast meat weight, and total yield. Carcass yield was calculated as eviscerated carcass weight relative to live weight before slaughter. The carcasses were cut as shown in Figure 1, and the breast, thigh, drumstick, and wing yields were calculated as their weight relative to eviscerated carcass weight. All parts were skin-on and bone-in after cut-up, except for the breast.

**FIGURE 2:**
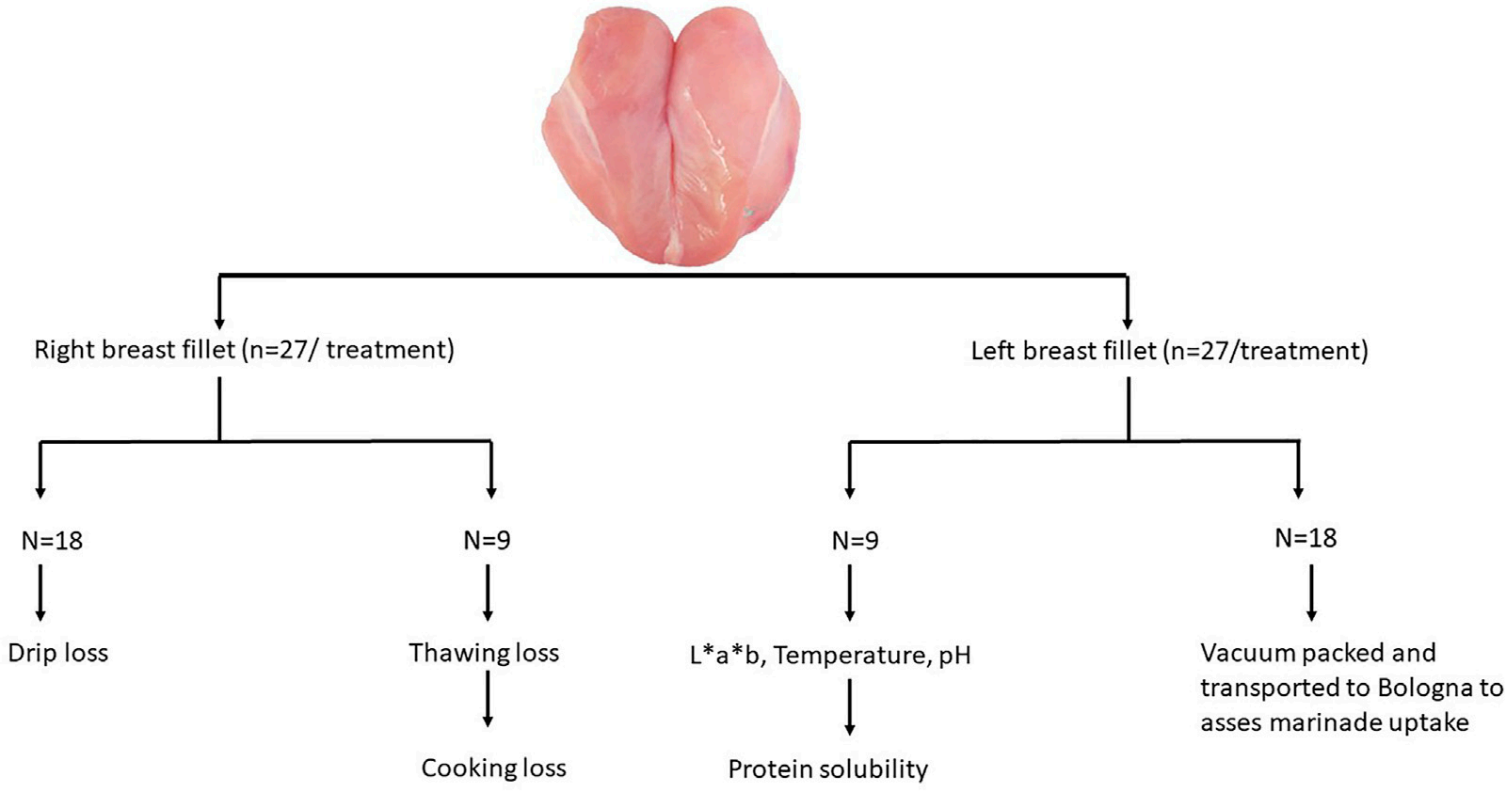
Overview of meat quality parameters determined on left and right breast fillet.

The different meat quality parameters were determined using the breast (pectoralis major muscles) (Figure 2). Left breast fillets (n = 9 per treatment) were weighed, and color measurements were performed using a MiniScan EZ colorimeter (Hunterlab, Reston, VA) to record CIE L* (lightness), a* (redness), and b* (yellowness) values. Temperature and pH ultimate were measured using a Portamess® 910 (Knick, Berlin, Germany). Following these measurements, breast fillets were vacuum-packed and stored at -20°C in order to determine protein solubility at a later time point. The remaining left breast fillets (n = 18 per treatment) were vacuum-packed and transported to the University of Bologna (Italy, Cesena, Department of Agricultural and Food Sciences) in order to determine marinade uptake. The right breast fillets (n = 18 per treatment) were removed from the carcass and put in a polypropylene bag, hung for 24 h at 4 ± 2°C, and then blotted dry, and weighed again to measure drip loss. Drip loss was calculated as the difference between the weight before storage (W1) and the weight after storage (W2) relative to the weight before storage. Drip loss was calculated using the following formula: drip loss (%) = [(W1–W2)/W1 ] × 100. The remaining right (n = 9 per treatment) breast fillets were weighed (W3), vacuum-packed, and stored at -20°C for 4 days. They were then defrosted for 24 h at 5°C, blotted dry, and weighed (W4) in order to determine thawing loss. Thawing loss was calculated using the following formula: thawing loss = [(W3–W4)/W3 ] × 100. After the thawing loss was determined, the fillets were cooked in a warm water bath (80°C) for 30 min. Afterward, they were blotted dry and weighed (W5) to record cooking loss. Cooking loss was calculated using the following formula: cooking loss (%) = [(W4—W5)/W4 ] × 100. Drip loss, thawing loss, and cooking loss were used to evaluate the water-holding capacity.

#### 2.2.1 Myofibrillar and sarcoplasmic protein solubility

Protein solubility was determined based on the difference in extractability of proteins in solutions at different ionic strengths. Sarcoplasmic protein solubility was determined by homogenizing (Ultraturrax, T25 basic, New Brunswick NJ) 3 g of minced meat in 80 ml of extraction medium (150 mM sodium chloride and 0.01 mM iodo acetic acid). The supernatant was centrifuged (3,000 g, 10 min) and filtered (Schleicher & Schuell nr. 597½), and the protein concentration of this supernatant was determined using the biuret method (Layne, 1957). To determine myofibrillar protein solubility, the remaining pellet was suspended in 45 ml of extraction buffer (0.1 M citric acid, 1 mM EDTA, 0.4 M sodium chloride, and 0.01 mM iodo acetic acid). The suspension was incubated at room temperature for 2 h. The supernatant was centrifuged (5,000 g, 20 min) and filtered (Schleicher & Schuell nr. 597½), and the protein concentration of this supernatant was determined using the biuret method (Layne, 1957).

#### 2.2.2 Marinade uptake

In order to assess marinade performances, meat was cut in order to obtain parallel cut samples (8 × 4 × 2 cm, weighing about 80 g), which were individually labeled and marinated by the addition of 20% marinade solution (6% sodium chloride and 1.8% sodium tripolyphosphate) using a small-scale vacuum tumbler (model MGH-20, Vakona Qualitat, Lienen, Germany). The tumbling time was 40 min under vacuum (-0.95 bar) (two working cycles of 20 min/cycle and one pause cycle of 5 min). After tumbling, samples were weighed again, and the difference in weight was used to determine marinade uptake. Marinade uptake was calculated based on carcass weight before marination (W1) and its weight after marination (W2), according to the following equation: marinade uptake (%) = [(W2 –W1)/W1] × 100.

### 2.3 Statistical analysis

Statistical analysis was performed in R for Windows (version 3.5.1). All data were checked for outliers and the normality of the residuals. Slaughter yield and meat quality were analyzed using a general linear model (GLM) with “zinc source” and “vitamin E level” as fixed factors and block as a random factor (factorial analysis). In the two-factorial analyses, when there was no significant interaction or no trend, only the main effects were taken into account. The differences were considered statistically significant at *p* < 0.05 and considered tendency at 0.05 < *p* < 0.1.

## 3 Results

### 3.1 Slaughter yield and meat quality

There were no interactions observed between the dietary zinc source and vitamin E level for total slaughter yield and the different meat quality parameters (Table 3). A tendency (*p* = 0.052) for live body weight was observed for the zinc source. Broilers that were fed a diet supplemented with ZnAA tended to have a higher slaughter weight than broilers that were fed diets supplemented with ZnSO_4._

**TABLE 3:** Effect of the supplemental zinc (Zn) source and vitamin E level (Vit E) on live weight and carcass composition of broilers at slaughter age (day 37).

Treatment	Live weight (g)	Breast weight (g)	Carcass yield (%)	Breast (%)	Drumstick (%)	Thigh (%)	Wing (%)
Zn source (Zn)							
1. ZnSO_4_	2,843	341.9	71.34	32.25	12.83	22.63	9.93
2. ZnAA	2,950	352.5	71.69	33.31	12.60	21.96	9.82
*p*-value	0.052	0.032	0.346	0.005	0.224	0.494	0.383
Vitamin E dose (E)							
1. 50 IU/kg	2,893	353.8	71.88	32.83	12.65	22.20	9.92
2. 100 IU/kg	2,913	340.7	71.15	32.73	12.79	22.39	9.84
*p*-value	0.677	0.807	0.098	0.768	0.425	0.750	0.572
Interaction Zn X E							
*p*-value	0.292	0.309	0.916	0.208	0.823	0.952	0.352
SEM	48.33	14.03	0.48	0.37	0.21	0.95	0.13

ZnAA, zinc amino acid complex; E, vitamin E; SEM, standard error of the mean.

Only a main effect of the zinc source on breast yield and certain meat quality parameters was observed, whereas no main effect of the vitamin E level was observed. The zinc source significantly affected the breast yield and water-holding capacity of the breast meat. A higher breast meat yield was observed for birds that fed a diet supplemented with ZnAA than birds that fed a diet supplemented with ZnSO_4_. Breast meat of birds that were fed a diet supplemented with ZnAA was characterized by a significantly lower drip loss and thawing loss than breast meat of birds that were fed a diet supplemented with ZnSO_4_. No effect of dietary treatment on cooking loss was observed. Dietary treatment did not significantly influence protein solubility and marinade uptake in breast meat (Table 4).

**TABLE 4:** Quality characteristics and functional properties of breast meat of broilers at slaughter age (day 37).

Treatment	pH	L*	a*	b*	Drip loss (%)	Thawing loss (%)	Cooking loss (%)	Marinade uptake (%)	Myofibrillar protein solubility (mg/ml)	Sarcoplasmic protein solubility (mg/ml)
Zn Source (Zn)										
1. ZnSO_4_	6.17	57.95	7.45	15.30	5.44	10.93	20.95	8.9	6.94	18.50
2. ZnAA	6.14	58.16	8.09	16.03	4.09	8.07	21.18	9.4	7.25	19.35
*p*-value	0.968	0.623	0.104	0.300	0.027	0.026	0.401	0.066	0.353	0.347
Vitamin E dose (E)										
1. 50 IU/kg	6.13	58.54	7.88	16.07	4.59	9.35	20.98	10.0	7.30	18.50
2. 100 IU/kg	6.18	57.67	7.66	15.26	4.94	9.66	21.15	8.3	6.90	18.38
*p*-value	0.685	0.858	0.782	0.353	0.582	0.819	0.765	0.052	0.244	0.236
Interaction Zn x E										
*p*-value	0.427	0.864	0.742	0.524	0.818	0.425	0.452	0.924	0.924	0.924
SEM	0.05	1.01	0.62	0.64	0.05	0.01	0.01	0.37	0.33	0.87

ZnAA, zinc amino acid complex; E, vitamin E; SEM, standard error of the mean; L*, lightness; a*, redness; b*, yellowness.

## 4 Discussion

Broilers reared under high temperatures often show lower meat yield and impaired meat quality (Zaboli et al., 2019). Breast meat of broilers exposed to chronic heat stress results in pale meat color (Petracci et al., 2004; Zhang et al., 2012), decreased WHC (Feng et al., 2008; Wang et al., 2009), and increased cook and drip losses (Woelfel et al., 2002) and is characterized by an increased denaturation of sarcoplasmic or myofibrillar proteins and a lower WHC (Zaboli et al., 2019). Additionally, higher breast meat yields are often associated with lower meat quality, characterized by a higher drip and cooking loss and lower marinade uptake (Petracci et al., 2015; Wang et al., 2017).

Interestingly, in the present study, it was observed that supplying zinc as ZnAA, resulted in increased yield in breast meat, characterized by lower drip and thawing losses, as compared to supplying zinc as ZnSO_4._ The lack of differences in cooking loss is probably due to the fact that fillets used to assess thawing loss were also used to assess the cooking loss. The increased breast yield might be partly attributed to the tendency for an increased live body weight of broilers supplemented with ZnAA, as modern broilers are selected for increased yield of pectoralis major muscles (Zuidhof et al., 2014). A previous study (De Grande et al., 2020) showed that ZnAA can improve performance under thermoneutral conditions. As zinc plays an important role in normal development and growth, it is possible that zinc supplements with increased bioavailability may better support growth under heat stress conditions. Although this could not be concluded from this study, no thermoneutral control group was included. Therefore, further research needs to be performed to confirm this hypothesis.

Zinc supplementation as such or increasing supplementation levels might decrease drip loss and improve the water-holding capacity of the meat under thermoneutral conditions (Liu et al., 2011; Yang et al., 2011). Trace minerals help to sustain the production in animals, improve nutrient utilization and at the same time effectively neutralize the oxidant stress and enhance the compromised immune system of heat-stressed birds (Mir et al., 2018). As the requirements for trace minerals increase during heat stress, the inclusion of a more readily available zinc source, such as ZnAA, might be more efficient in reducing the adverse effects of heat stress on meat quality (Sahin et al., 2009; Chand et al., 2014; Farag and Alagawany, 2018). In addition, Liu et al. (2015) reported that increased dietary supplementation of Zn can upregulate the expression of Zn-containing superoxide dismutase. As the negative impact of high ambient temperatures on meat quality is mainly caused by oxidative damage to the skeletal muscle (Zaboli et al., 2019), the improved quality traits when supplying ZnAA could be ascribed to improved support of the antioxidant defense system (Liu et al., 2015; Kamran Azad et al., 2018). Indeed, a previous study performed by De Grande et al. (2020) showed that ZnAA could decrease the activity of the glutathione peroxidase in plasma on day 36, while malondialdehyde levels did not differ, indicating that ZnAA might better support the oxidative status.

Although it has been acknowledged that vitamin E has a positive effect on meat quality by protecting membranes against lipid oxidation, thus reducing drip loss in meat (Estevez, 2015; Pompeu et al., 2018), no effects could be observed when the dietary vitamin E level was increased in the present study. It is possible that the increase in the level of vitamin E was insufficient to create an impact on meat quality and yield under these conditions; in the recent literature, supplementation at a level of 250 mg/kg was advised to improve meat quality in broiler chickens (Shakeri et al., 2020).

Overall, it can be argued that an organic form of Zn, in particular ZnAA, which is characterized by improved bioavailability (Star et al., 2012; De Grande et al., 2020), might be able to better mitigate lipid and protein oxidation in post-rigor breast muscles and increase both the water-holding capacity and water-binding ability. However, as no thermoneutral control group was incorporated in this study, the observed effects of the zinc source cannot be generalized as a solution for the negative effects of heat stress. Therefore, further research needs to be performed to elucidate the underlying mechanism concerning the effects of zinc sources on meat yield and quality.

In conclusion, comparing ZnSO_4_ with more readily available ZnAA shows improved breast meat yield and increased water-holding capacity in broilers exposed to chronic cyclic heat stress at the end of the production cycle. Moreover, the beneficial effects of ZnAA on breast meat yield and quality seem to be independent of the vitamin E level, and increasing the vitamin E level has no additional beneficial effects.

## Data Availability

The raw data supporting the conclusion of this article will be made available by the authors, without undue reservation.
